# Mediastinal cryptococcoma as part of disseminated cryptococcosis in HIV-negative pregnant woman with Myasthenia Gravis: Autopsy case report

**DOI:** 10.1016/j.mmcr.2025.100726

**Published:** 2025-08-20

**Authors:** Bianca Ponnamma Samy, Nosipho Maria Thobakgale, Moshawa Calvin Khaba

**Affiliations:** Department of Anatomical Pathology, Dr George Mukhari Academic Laboratory, National Health Laboratory Service, Sefako Makgatho Health Sciences University, South Africa

**Keywords:** Myasthenia gravis, Cryptococcosis, Pregnancy, HIV/AIDS, Autopsy

## Abstract

Cryptococcosis is a fungal infection commonly found in immunocompromised individuals, but it can also affect immunocompetent individuals. A pregnant woman with myasthenia gravis, HIV-negative, died after a diagnosis of tuberculosis. An autopsy revealed a mediastinal mass and organ involvement, indicating a disseminated cryptococcal infection. This case highlights the importance of maintaining a high index of suspicion for cryptococcal infection in immunosuppressed individuals, even without HIV/AIDS. Early detection is achieved by measuring serum cryptococcal antigen.

## 1 Introduction

Cryptococcosis is a fungal infection caused by the fungi *Cryptococcus* that consists of two species, such as *Cryptococcus neoformans* and *Cryptococcus gattii,* which are commonly associated with human disease [[1], [2], [3]]. Cryptococcal infection is mostly prevalent in immunocompromised people, particularly those with human immunodeficiency virus (HIV) infection, cancer, organ transplant recipients, and people who use corticosteroids on a regular or chronic basis. It can, however, be seen in immunocompetent individuals, although the index of suspicion in such cases for infection is lower due to its uncommon occurrence [4,5].

Myasthenia gravis (MG) is a chronic antibody-mediated autoimmune disorder that affects the neuromuscular junctions [6,7]. MG is characterised by muscle weakness due to impaired function of the acetylcholine (ACh) receptors at the neuromuscular junction from autoantibodies acting against these receptors. Hyperplasia and tumours of the thymus also contribute to increased production of the autoantibodies [6]. Thymic tumours are frequently associated with mediastinal masses in MG, with an estimated 10–20 % of patients with myasthenia gravis being diagnosed with thymoma.

While there is no evidence that MG can adversely affect pregnancy outcomes, individuals during pregnancy can present with symptoms of a myasthenic crisis, including respiratory distress and excessive fatigue. In some cases of pregnancy and MG, individuals may go into remission; however, pregnancy overall does not appear to worsen the disease. Immune suppression caused by MG treatment can compromise immune function, increasing susceptibility to infections, including opportunistic infections [6]. With this in mind, cryptococcosis has been reported in MG only infrequently, particularly in pregnant patients. While corticosteroid treatment for MG may cause immunosuppression, it is unclear whether pregnancy also contributes to immunosuppression and increases the risk of infection in this case. According to a search of the literature, this combination has never been reported before. The aim of this study is to raise awareness of this combination so the clinicopathological team may high index of suspicion is unsuspecting cases.

## 2 Case presentation

### 2.1 Clinical presentation

We report the case of a 29-year-old pregnant woman who was HIV negative at 18 weeks’ gestational age, who was diagnosed with Myastenia Gravis (MG) 3 years ago.

She was treated with pyridostigmine 60mg three times daily and Dexamethasone, initially 10mg once off, then tapered down to 4mg per os three times daily for MG.

She had previously experienced respiratory distress symptoms a year prior to her demise, when she was admitted and diagnosed with pulmonary tuberculosis. She was treated with rifafour (rifampicin 150 mg, isoniazid 75 mg, pyrazinamide 400 mg and ethambutol hydrochloride 275 mg) 4 tablets daily, and upon showing clinical improvement, she was subsequently discharged to continue with TB treatment at the local clinic. On this index presentation, she presented with dyspnoea, generalised weakness, headache, diplopia and ear congestion. On general examination, she was chronically ill, emaciated and dehydrated with bipedal oedema. She had a blood pressure of 122/85 mmHg with a heart rate of 102 beats per minute. She had a fever of 38.7 °C, and was tachypnoeic with a saturation of 95 % on 40 % nasal prong oxygen. The lung assessment revealed bilateral crackles with decreased air entry. There were audible and normal heartbeats. The abdomen was soft and non-tender. A normal Glasgow Coma Score (GCS) of 15/15, cranial nerve III palsy, and bilateral ptosis were discovered during the neurological examination. There was no evidence of meningitis.

**Fig. 1:**
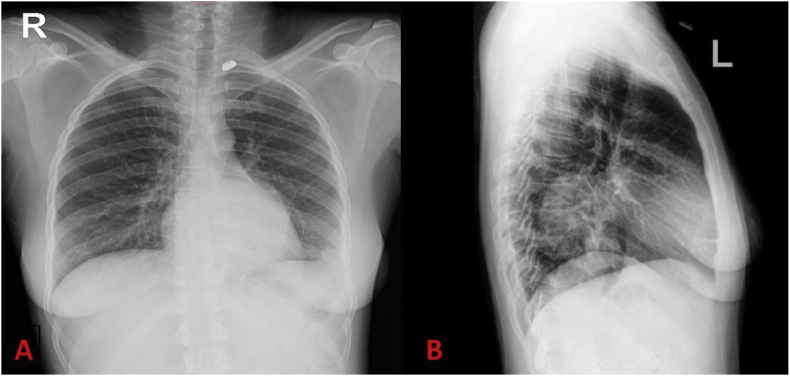
A. Antero-lateral view, B. Lateral view - shows bilateral hilar lymphadenopathy and bilateral infiltrates on a chest x-ray.

At this point, the chest x-ray revealed bilateral hilar lymphadenopathy (Fig. 1). The nasopharyngeal swab was negative for SARS-CoV-2, and the full blood count showed a leucocytosis with normal haemoglobin, whilst the kidney function showed pre-renal failure and metabolic acidosis. The erythrocyte sedimentation rate (ESR) and C-reactive protein (CRP) levels were both significantly elevated, at more than 120mm/hr and 74mg/L, respectively.

The blood submitted for serum cryptococcal antigen was insufficient and not repeated; the reason for this is unknown.

Cerebrospinal fluid revealed low adenosine deaminase levels in the absence of growth, and a cysticercosis enzyme-linked immunosorbent assay (ELISA) test was negative.

The computed tomography (CT) and magnetic resonance imaging (MRI) showed multiple ring enhancing lesions on the pons, occipital and temporal areas.

While pulmonary tuberculosis was still the working diagnosis based on the diagnosis made in the previous year, based on the blood and radiological investigations, other differential diagnoses, that may present with similar imaging features, such as mass effect or space-occupying lesions in the central nervous system, such as metastatic diseases. Other infectious diseases, such as central nervous system tuberculoma and toxoplasmosis, were also considered. To differentiate these conditions, a combination of clinical features, radiological imaging and microbiological studies is crucial.

On admission, the patient was started on ceftriaxone 1g intravenous every 12 hourly, metronidazole 500mg per os (po) 12 hourly, augmentin (amoxicillin 250mg, clavulanic acid 125mg) 2 tablets po 12 hourly and bactrim (trimethoprim 800mg/sulfamethoxazole 160mg) 4 tablets po 6 hourly.

However, her clinical condition deteriorated during this index admission, and she died in the ward. Based on the diagnostic conundrum of this case, an autopsy was requested.

### 2.2 Autopsy findings

The autopsy was carried out under strict biosafety guidelines, with permission and consent from the family.

### 2.3 Gross features

The body mass index of the deceased was 18.7 kg/m2. She was emaciated, hypovolaemic, and with pale mucosa. Serous bilateral pleural effusion and ascites were noted.

Both lungs were increased in weight. They were oedematous, friable with a pale, hemorrhagic nodule that measured 10 × 5mm.

**Fig. 2:**
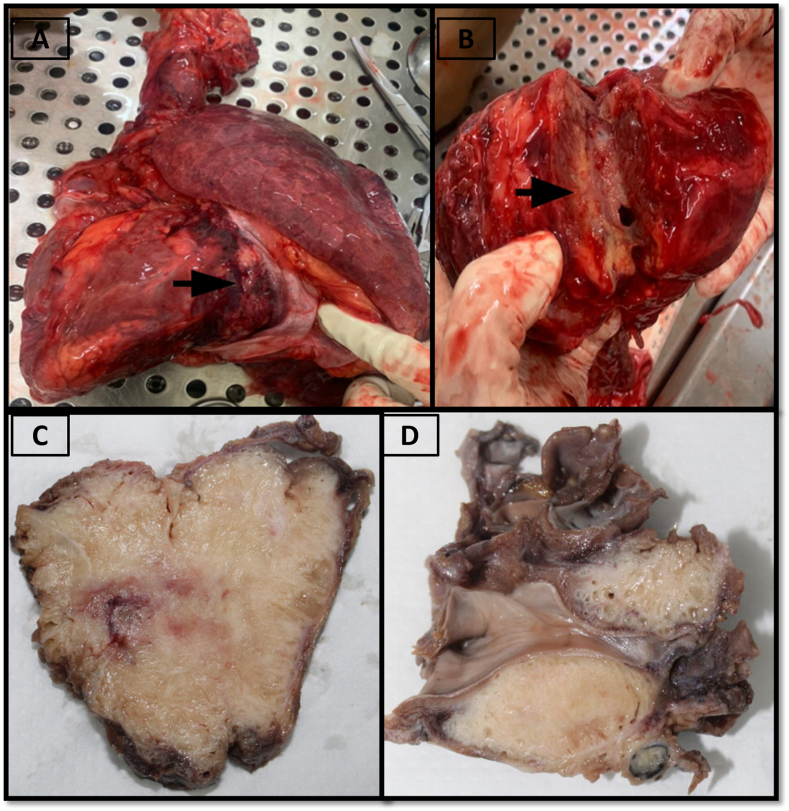
A and B - Fresh dissection of heart with mass attached; cut surface was soft, white-tan, and gelatinous. C and D – Post formalin fixation of heart; cut surface shows a mass with sponge to cystic texture with pale tan appearance.

A mass attached to the heart measuring 70 x 60 × 30mm was noted. It was connected to the left atrium and encased major blood vessels. The cut surface was soft, white-tan, spongy and gelatinous. It constricted the left atrial cavity (Fig. 2).

The liver and kidneys had isolated pale nodules measuring 3–5mm. The brain showed signs of increased intracranial pressure. Multiple white-tan lesions involving the brainstem, cerebellum, temporal and frontal cortex were visible on the cut surface. The sizes of these lesions ranged from 5 × 5mm to 10 x 10mm. The meninges were exudate-free. The rest of the organs were unremarkable, and pregnancy was confirmed.

### 2.4 Microscopic findings

**Fig. 3:**
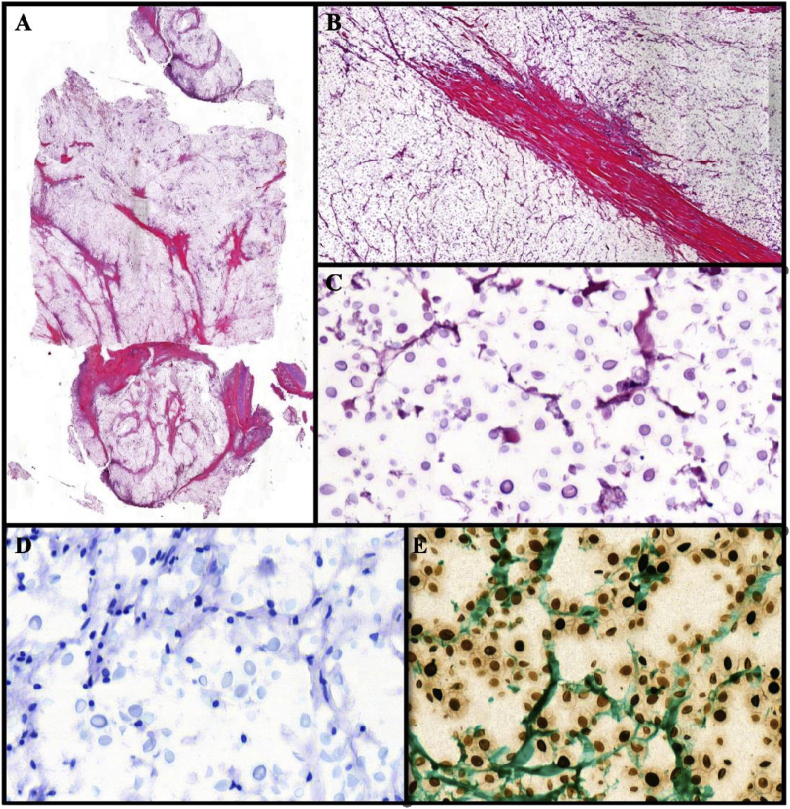
A (H&E): Mediastinal mass with fungal yeast infiltrate. B (H&E): soap bubble appearance, consistent with cryptococcal infection. C (PAS) and D fungi ranged in size from round to and encapsulated with narrow neck budding; ranged in size from 4 to 10 μm and capsule was highlighted by mucicarmine. E − fungi highlighted by groccot

Sections of the mediastinal mass revealed a fungal yeast infiltrate with a soap bubble appearance, consistent with cryptococcal infection. The fungal yeasts ranged in size from round to oval. They were encapsulated with narrow neck budding and ranged in size from 4 to 10 μm. These fungi were highlighted by Gomori Methenamine silver and Periodic Acid Schiff (PAS) (Fig. 3), while the capsule was highlighted by mucicarmine. The hypophysis, brain stem, cerebellum, right frontal cortex, bone marrow, pancreas, kidneys, and lungs were also involved. Furthermore, pneumonic changes were observed in the lungs.

Disseminated cryptococcal infection was the final cause of death.

## 3 Discussion

Cryptococcal infection is a common opportunistic infection in immunocompromised individuals, particularly in our setting, which is an HIV/AIDS hotspot. However, it has been observed in immunocompetent individuals. In most cases, clinical and radiological features are insufficient to make a diagnosis. It is frequently misdiagnosed as pulmonary tuberculosis (TB) or *pneumocystis jiroveci* infection. Microscopic examination of clinical specimens submitted for microbiological or histopathological identification of the fungi confirms the definitive diagnosis [2,4,8]. The most sensitive way to diagnose this infection is with cryptococcal antigen positivity in cerebrospinal fluid and/or serum [2].

The non-specific clinical and radiological features in this case were exacerbated by the fact that the descendant showed some clinical improvement after starting anti-TB therapy.

This could also be explained by the fact that tuberculosis is endemic in our area, so it was considered the most likely diagnosis at first. However, because the deceased was HIV negative, national TB diagnostic guidelines must be followed at all times.

In this case, it was imperative to reassess the earlier tuberculosis diagnosis; yet, the administration of anti-TB treatment persisted. The initial clinical improvement on anti-TB treatment in the context of steroid therapy may potentially be perceived as a form of masking symptoms rather than a true clinical improvement. Despite testing negative for HIV, the patient's prolonged use of steroid medication for MG could weaken her immune system, making her more susceptible to opportunistic infections, including tuberculosis and cryptococcosis. It is therefore important to continually maintain a high index of suspicion for opportunistic infection in this context.

Importantly, the blood submitted for cryptococcal antigen testing could have resulted in a timely diagnosis for this lethal infection. Nevertheless, in this case, it proved to be insufficient, albeit the reason for not repeating it remains unexplained.

Cryptococcal infection has only been reported in a few cases of myasthenia gravis patients.

MG is an autoimmune disease with a poor prognosis despite immunosuppressive therapy. In MG, autoantibodies attack specific receptors on the surface of muscle cells, preventing acetylcholine from binding to them and, as a result, preventing the muscle from responding to a nerve signal. Thymic pathology, such as thymic hyperplasia or thymoma, has been linked to MG. This is why the combination of immunosuppressive therapy and thymic gland surgical removal has improved the clinical outcome of MG [9].

It's not surprising that the first macroscopic differential diagnosis of the mass attached to the heart during the autopsy procedure was a thymoma. Further microscopic examination, however, confirmed cryptococcoma. Although the index patient tested negative for HIV, her immune system was compromised by the immunosuppressive treatment she was receiving for myasthenia, making her more vulnerable to opportunistic infections like this one.

It remains to be seen whether her pregnancy weakened her already compromised immune system. Although this is still debated, there are several physiological immune system adjustments that occur during pregnancy that are specifically designed to prevent rejection of the antigenically different fetus. Furthermore, lymphocyte proliferation is reduced, including the pooling of helper T-cell and natural killer cell activity [10].

Further to that, the altered hormonal status aggravates the already compromised cell-mediated immune response.

The pregnant woman is more vulnerable to microorganisms that require a cell-mediated immune response, such as fungal organisms, due to the physiological depletion in maternal cell-mediated immunity. As a result, cryptococcal infection in pregnant women is extremely dangerous [10].

In summary, this case emphasises the importance of maintaining a high index of suspicion for cryptococcal infection in unsuspecting cases of immunosuppression from other causes, particularly in the absence of HIV/AIDS. In these cases, serum cryptococcal antigen is the most straightforward way to reach an early diagnosis.

## CRediT authorship contribution statement

**Bianca Ponnamma Samy:** Writing – review & editing, Writing – original draft, Conceptualization. **Nosipho Maria Thobakgale:** Writing – review & editing, Writing – original draft, Conceptualization. **Moshawa Calvin Khaba:** Writing – review & editing, Writing – original draft, Conceptualization.

## Consent

Written informed consent was obtained from the patient or legal guardian(s) for publication of this case report and accompanying images. A copy of the written consent is available for review by the Editor-in-Chief of this journal on request.

## Funding source

There are none.

## Conflict of interest

There are none.
